# Supplementary material for: A Likelihood Approach for Real-Time Calibration of Stochastic Compartmental Epidemic Models
Source: PLoS Comput Biol. 2017 Jan 17;13(1):e1005257. doi: 10.1371/journal.pcbi.1005257 (PMC5240920; doi:10.1371/journal.pcbi.1005257)
Supplement: S1 File — (TAR.GZ) [file pcbi.1005257.s014.tar.gz › HSPH_Online-SI-Revision/MSS10/n1-mild/n1-mild_NEW2_table8.pdf]

1	newly 3 weeks spec	9.	6.41	{0., 15.}	64.1111	0.661096	newly 3 weeks cumul	15.	22.75	{4., 45.}	85.4	0.313697	newly 5 weeks spec	11.	3.59	{0., 11.}	77.	1.15247	newly 5 weeks cumul	33.	31.24	{4., 59.}	56.7273	0.341229	newly 47 weeks cumul	75.	40.54	{4., 83.}	60.0267	0.520295
2	newly 3 weeks spec	1.	1.84	{0., 6.}	222.	0.88244	newly 3 weeks cumul	7.	8.66	{1., 18.}	88.	0.381159	newly 5 weeks spec	1.	0.72	{0., 2.}	146.	0.941313	newly 5 weeks cumul	9.	10.43	{1., 27.}	94.5556	0.440997	newly 43 weeks cumul	9.	10.86	{1., 27.}	99.3333	0.444599
3	newly 3 weeks spec	7.	8.58	{0., 27.}	116.857	0.909909	newly 3 weeks cumul	17.	41.75	{8., 84.}	168.765	0.410282	newly 5 weeks spec	1.	3.31	{0., 8.}	357.	0.915093	newly 5 weeks cumul	22.	50.67	{8., 108.}	162.409	0.422531	newly 52 weeks cumul	22.	53.89	{8., 118.}	177.045	0.437276
4	newly 3 weeks spec	14.	8.08	{0., 19.}	77.5714	1.00541	newly 3 weeks cumul	32.	31.37	{4., 70.}	67.7813	0.380455	newly 5 weeks spec	6.	4.08	{0., 19.}	112.	1.27304	newly 5 weeks cumul	48.	41.73	{4., 91.}	69.5208	0.448551	newly 46 weeks cumul	263.	55.	{4., 147.}	79.2776	0.974602
5	newly 3 weeks spec	5.	3.28	{0., 9.}	96.4	1.07374	newly 3 weeks cumul	17.	15.93	{3., 29.}	54.1765	0.295078	newly 5 weeks spec	1.	0.55	{0., 1.}	131.	0.941918	newly 5 weeks cumul	20.	17.64	{3., 33.}	58.4	0.337106	newly 48 weeks cumul	23.	18.25	{3., 39.}	58.8261	0.36741
6	newly 3 weeks spec	3.	8.73	{0., 20.}	249.	0.757222	newly 3 weeks cumul	20.	41.73	{10., 79.}	133.35	0.371617	newly 5 weeks spec	7.	4.78	{0., 16.}	89.7143	1.07622	newly 5 weeks cumul	35.	52.48	{10., 102.}	90.6286	0.349451	newly 52 weeks cumul	116.	60.33	{10., 121.}	55.4052	0.49025
7	newly 3 weeks spec	5.	2.66	{0., 9.}	104.	1.27364	newly 3 weeks cumul	17.	34.97	{4., 71.}	135.588	0.393256	newly 5 weeks spec	6.	0.02	{0., 0.}	99.6667	1.75815	newly 5 weeks cumul	25.	35.49	{4., 71.}	85.8	0.351195	newly 51 weeks cumul	45.	35.49	{4., 71.}	54.8667	0.38533
8	newly 3 weeks spec	13.	8.77	{1., 17.}	57.1538	0.45532	newly 3 weeks cumul	40.	34.35	{14., 62.}	38.625	0.220897	newly 5 weeks spec	15.	5.04	{0., 14.}	71.6	0.990359	newly 5 weeks cumul	63.	46.08	{16., 90.}	44.8254	0.289338	newly 43 weeks cumul	262.	68.25	{17., 167.}	73.9504	0.740293
9	newly 3 weeks spec	0.1	4.11	{0., 12.}	4096.	0.967756	newly 3 weeks cumul	7.	18.89	{2., 36.}	197.286	0.462191	newly 5 weeks spec	0.1	1.08	{0., 5.}	1132.	0.367496	newly 5 weeks cumul	7.	22.4	{2., 42.}	247.429	0.509604	newly 42 weeks cumul	7.	23.88	{2., 50.}	268.571	0.52192
10	newly 3 weeks spec	0.1	3.73	{0., 10.}	3674.	1.20707	newly 3 weeks cumul	1.	12.57	{4., 24.}	1157.	1.00493	newly 5 weeks spec	0.1	1.87	{0., 6.}	1874.	0.709512	newly 5 weeks cumul	1.	16.95	{4., 34.}	1595.	1.10352	newly 43 weeks cumul	1.	23.75	{4., 54.}	2275.	1.19021
11	newly 3 weeks spec	14.	6.13	{0., 14.}	66.9286	0.853604	newly 3 weeks cumul	36.	23.76	{6., 46.}	49.4444	0.357329	newly 5 weeks spec	13.	4.74	{0., 12.}	78.3077	1.15501	newly 5 weeks cumul	55.	33.94	{6., 75.}	56.8727	0.433455	newly 42 weeks cumul	283.	49.35	{6., 126.}	82.5618	0.988127
12	newly 3 weeks spec	13.	6.34	{0., 24.}	90.	1.38704	newly 3 weeks cumul	24.	29.08	{3., 67.}	91.25	0.440408	newly 5 weeks spec	5.	2.94	{0., 11.}	126.4	1.49242	newly 5 weeks cumul	44.	36.14	{3., 91.}	77.9091	0.547804	newly 43 weeks cumul	158.	42.49	{3., 125.}	75.6013	0.925749
13	newly 3 weeks spec	2.	2.18	{0., 6.}	105.	0.701505	newly 3 weeks cumul	5.	9.22	{2., 19.}	108.	0.303872	newly 5 weeks spec	0.1	1.09	{0., 4.}	1126.	0.447126	newly 5 weeks cumul	5.	11.92	{2., 28.}	159.6	0.36859	newly 44 weeks cumul	5.	15.59	{2., 36.}	233.	0.414538
14	newly 3 weeks spec	0.1	2.78	{0., 8.}	2766.	0.885829	newly 3 weeks cumul	3.	12.4	{3., 25.}	318.667	0.529916	newly 5 weeks spec	0.1	1.25	{0., 5.}	1288.	0.443465	newly 5 weeks cumul	3.	15.49	{3., 32.}	421.667	0.587191	newly 48 weeks cumul	3.	16.9	{3., 37.}	468.667	0.606161
15	newly 3 weeks spec	14.	5.74	{0., 15.}	68.4286	0.890471	newly 3 weeks cumul	34.	27.32	{6., 50.}	51.	0.315013	newly 5 weeks spec	7.	1.94	{0., 8.}	88.2857	1.3652	newly 5 weeks cumul	49.	32.24	{6., 66.}	54.9388	0.390034	newly 48 weeks cumul	104.	34.56	{6., 66.}	69.3462	0.648811
16	newly 3 weeks spec	6.	9.87	{0., 22.}	113.833	0.492736	newly 3 weeks cumul	34.	39.66	{11., 67.}	59.8235	0.250122	newly 5 weeks spec	9.	4.97	{0., 12.}	73.6667	0.920267	newly 5 weeks cumul	52.	51.31	{12., 99.}	54.2885	0.283273	newly 46 weeks cumul	164.	65.29	{12., 131.}	61.2378	0.556511
17	newly 3 weeks spec	12.	8.56	{0., 20.}	60.5	0.597609	newly 3 weeks cumul	46.	29.37	{8., 57.}	48.2391	0.346276	newly 5 weeks spec	14.	6.42	{0., 16.}	64.2857	0.87889	newly 5 weeks cumul	71.	42.65	{8., 89.}	50.8592	0.397174	newly 49 weeks cumul	150.	65.45	{8., 148.}	60.0467	0.572311
18	newly 3 weeks spec	9.	7.27	{0., 15.}	60.1111	0.491151	newly 3 weeks cumul	36.	28.18	{9., 49.}	43.6111	0.267218	newly 5 weeks spec	12.	3.7	{0., 11.}	76.3333	1.1067	newly 5 weeks cumul	56.	37.13	{10., 69.}	46.3393	0.320949	newly 42 weeks cumul	105.	48.86	{11., 105.}	59.2952	0.483699
19	newly 3 weeks spec	9.	4.72	{0., 14.}	72.	0.922304	newly 3 weeks cumul	31.	24.17	{5., 46.}	48.0323	0.331976	newly 5 weeks spec	2.	1.72	{0., 6.}	130.	1.04612	newly 5 weeks cumul	38.	28.87	{5., 60.}	53.6579	0.379756	newly 49 weeks cumul	38.	30.43	{5., 65.}	57.0789	0.388799
20	newly 3 weeks spec	2.	4.7	{0., 14.}	216.	0.778719	newly 3 weeks cumul	25.	20.44	{4., 40.}	51.44	0.311749	newly 5 weeks spec	0.1	2.09	{0., 8.}	2132.	0.488603	newly 5 weeks cumul	27.	25.22	{4., 53.}	61.1852	0.338017	newly 46 weeks cumul	27.	28.71	{4., 64.}	73.0741	0.361299
21	newly 3 weeks spec	2.	2.84	{0., 12.}	166.	0.955309	newly 3 weeks cumul	9.	19.55	{2., 43.}	154.556	0.416139	newly 5 weeks spec	1.	0.71	{0., 0.}	157.	0.998907	newly 5 weeks cumul	10.	21.58	{2., 53.}	157.	0.42535	newly 46 weeks cumul	10.	22.23	{2., 54.}	163.5	0.429866
22	newly 3 weeks spec	11.	19.98	{9., 31.}	93.0909	0.297363	newly 3 weeks cumul	38.	63.07	{37., 83.}	72.2368	0.234021	newly 5 weeks spec	8.	15.91	{5., 27.}	116.375	0.366749	newly 5 weeks cumul	55.	96.94	{49., 134.}	83.5273	0.262779	newly 48 weeks cumul	274.	196.64	{92., 284.}	30.1679	0.200605
23	newly 3 weeks spec	4.	7.26	{0., 21.}	157.	0.734222	newly 3 weeks cumul	24.	36.58	{6., 79.}	88.75	0.332122	newly 5 weeks spec	9.	3.29	{0., 11.}	84.1111	1.29179	newly 5 weeks cumul	45.	45.66	{6., 102.}	64.3111	0.368936	newly 43 weeks cumul	68.	50.58	{6., 115.}	59.6471	0.442175
24	newly 3 weeks spec	9.	8.86	{1., 18.}	61.3333	0.431316	newly 3 weeks cumul	31.	33.75	{9., 57.}	44.6774	0.230796	newly 5 weeks spec	8.	5.26	{0., 13.}	65.25	0.700331	newly 5 weeks cumul	48.	46.06	{10., 76.}	43.0833	0.257168	newly 45 weeks cumul	112.	63.85	{10., 122.}	49.6875	0.411853
25	newly 3 weeks spec	8.	6.16	{0., 15.}	67.25	0.636344	newly 3 weeks cumul	29.	21.19	{6., 37.}	45.7586	0.301123	newly 5 weeks spec	12.	3.87	{0., 11.}	74.5833	1.13929	newly 5 weeks cumul	48.	29.72	{6., 61.}	50.7917	0.384063	newly 42 weeks cumul	155.	41.62	{6., 98.}	73.1742	0.76154
26	newly 3 weeks spec	3.	4.57	{0., 11.}	123.	0.635363	newly 3 weeks cumul	7.	16.73	{4., 33.}	159.286	0.379536	newly 5 weeks spec	0.1	2.65	{0., 10.}	2642.	0.830367	newly 5 weeks cumul	11.	23.33	{4., 46.}	139.909	0.372925	newly 45 weeks cumul	11.	33.52	{4., 85.}	231.273	0.441967
27	newly 3 weeks spec	13.	7.09	{0., 19.}	74.3846	0.954453	newly 3 weeks cumul	30.	33.22	{4., 66.}	70.6667	0.370503	newly 5 weeks spec	5.	3.43	{0., 12.}	107.4	1.19414	newly 5 weeks cumul	42.	41.39	{4., 89.}	69.8333	0.420639	newly 42 weeks cumul	51.	46.26	{4., 111.}	69.8431	0.455755
28	newly 3 weeks spec	5.	14.69	{0., 38.}	241.4	0.6964	newly 3 weeks cumul	18.	45.1	{6., 96.}	178.778	0.436005	newly 5 weeks spec	9.	10.71	{0., 26.}	106.778	0.896039	newly 5 weeks cumul	31.	69.45	{7., 159.}	160.032	0.443726	newly 48 weeks cumul	190.	97.42	{7., 225.}	58.8316	0.596936
29	newly 3 weeks spec	0.1	4.01	{0., 12.}	3998.	0.963529	newly 3 weeks cumul	5.	20.4	{4., 41.}	316.8	0.537528	newly 5 weeks spec	0.1	1.19	{0., 4.}	1238.	0.391008	newly 5 weeks cumul	5.	23.66	{4., 48.}	382.	0.58215	newly 45 weeks cumul	5.	24.98	{4., 50.}	408.4	0.593942
30	newly 3 weeks spec	8.	7.46	{0., 16.}	69.25	0.538269	newly 3 weeks cumul	27.	28.85	{8., 52.}	49.5926	0.248261	newly 5 weeks spec	5.	3.75	{0., 10.}	90.2	0.879912	newly 5 weeks cumul	40.	37.47	{9., 68.}	49.125	0.282524	newly 49 weeks cumul	44.	47.76	{9., 98.}	63.0455	0.320815
31	newly 3 weeks spec	0.1	2.84	{0., 7.}	2796.	1.0631	newly 3 weeks cumul	0.1	11.7	{3., 22.}	11600.	1.96619	newly 5 weeks spec	0.1	1.65	{0., 6.}	1670.	0.587708	newly 5 weeks cumul	0.1	15.58	{3., 32.}	15480.	2.04958	newly 47 weeks cumul	0.1	17.66	{3., 37.}	17560.	2.07892
32	newly 3 weeks spec	17.	11.23	{0., 28.}	75.2353	0.929488	newly 3 weeks cumul	43.	49.33	{11., 101.}	67.9302	0.318911	newly 5 weeks spec	10.	7.39	{0., 27.}	99.3	1.22697	newly 5 weeks cumul	62.	65.63	{11., 154.}	70.371	0.370712	newly 45 weeks cumul	224.	89.26	{11., 213.}	62.9375	0.649137
33	newly 3 weeks spec	3.	7.14	{1., 13.}	158.667	0.425414	newly 3 weeks cumul	15.	26.32	{13., 40.}	83.3333	0.247642	newly 5 weeks spec	1.	4.58	{0., 10.}	394.	0.694286	newly 5 weeks cumul	19.	36.45	{15., 60.}	101.737	0.290404	newly 46 weeks cumul	20.	50.56	{16., 86.}	162.8	0.384161
34	newly 3 weeks spec	9.	7.27	{0., 17.}	83.	0.755605	newly 3 weeks cumul	32.	26.32	{8., 48.}	51.375	0.302317	newly 5 weeks spec	6.	4.57	{0., 14.}	97.1667	1.00506	newly 5 weeks cumul	42.	37.18	{8., 79.}	59.3333	0.342711	newly 45 weeks cumul	93.	48.83	{8., 113.}	61.8387	0.521903
35	newly 3 weeks spec	10.	13.13	{7., 21.}	49.9	0.185776	newly 3 weeks cumul	37.	53.05	{37., 69.}	46.7838	0.160504	newly 5 weeks spec	4.	8.37	{2., 15.}	128.25	0.365581	newly 5 weeks cumul	48.	71.75	{47., 91.}	53.2292	0.178555	newly 47 weeks cumul	53.	97.19	{54., 137.}	86.6981	0.253703
36	newly 3 weeks spec	0.1	1.99	{0., 5.}	2006.	0.625099	newly 3 weeks cumul	0.1	10.45	{3., 23.}	10350.	1.873	newly 5 weeks spec	0.1	0.63	{0., 1.}	702.	0.20851	newly 5 weeks cumul	0.1	12.19	{3., 28.}	12090.	1.90414	newly 49 weeks cumul	0.1	13.31	{3., 36.}	13210.	1.91579
37	newly 3 weeks spec	5.	6.6	{0., 18.}	110.	0.712375	newly 3 weeks cumul	27.	27.78	{4., 60.}	60.6667	0.314245	newly 5 weeks spec	7.	3.09	{0., 12.}	91.5714	1.3012	newly 5 weeks cumul	39.	34.64	{4., 82.}	61.1282	0.371786	newly 45 weeks cumul	50.	40.85	{4., 95.}	66.14	0.435451
38	newly 3 weeks spec	2.	3.29	{0., 9.}	167.5	0.848907	newly 3 weeks cumul	11.	18.18	{3., 37.}	101.091	0.343139	newly 5 weeks spec	0.1	1.4	{0., 4.}	1452.	0.383141	newly 5 weeks cumul	12.	21.59	{3., 47.}	117.917	0.366014	newly 49 weeks cumul	12.	24.17	{3., 60.}	139.417	0.384601
39	newly 3 weeks spec	4.	4.39	{0., 13.}	107.25	0.778538	newly 3 weeks cumul	11.	19.03	{3., 37.}	102.818	0.332215	newly 5 weeks spec	4.	2.28	{0., 8.}	102.	1.18921	newly 5 weeks cumul	27.	23.91	{3., 49.}	62.9259	0.384625	newly 47 weeks cumul	33.	27.55	{3., 62.}	68.5758	0.442292
40	newly 3 weeks spec	0.1	3.13	{0., 11.}	3134.	0.774535	newly 3 weeks cumul	1.	15.74	{3., 35.}	1474.	1.01201	newly 5 weeks spec	0.1	1.01	{0., 3.}	1074.	0.280625	newly 5 weeks cumul	1.	18.51	{3., 49.}	1751.	1.04515	newly 50 weeks cumul	1.	20.13	{3., 52.}	1913.	1.05867
41	newly 3 weeks spec	3.	2.36	{0., 9.}	120.667	1.12437	newly 3 weeks cumul	11.	23.22	{3., 51.}	148.727	0.408807	newly 5 weeks spec	0.1	0.67	{0., 0.}	756.	0.125544	newly 5 weeks cumul	11.	24.98	{3., 62.}	164.727	0.421041	newly 51 weeks cumul	11.	26.18	{3., 65.}	175.636	0.428068
42	newly 3 weeks spec	0.1	3.07	{0., 10.}	3106.	0.568946	newly 3 weeks cumul	0.1	18.87	{3., 36.}	18770.	2.07421	newly 5 weeks spec	0.1	1.05	{0., 0.}	1130.	0.179896	newly 5 weeks cumul	0.1	21.19	{3., 60.}	21090.	2.09134	newly 50 weeks cumul	0.1	21.76	{3., 60.}	21660.	2.09399
43	newly 3 weeks spec	0.1	1.35	{0., 4.}	1392.	0.438589	newly 3 weeks cumul	5.	7.61	{2., 16.}	96.2	0.318627	newly 5 weeks spec	0.1	0.37	{0., 0.}	454.	0.123167	newly 5 weeks cumul	5.	8.59	{2., 18.}	113.8	0.334732	newly 52 weeks cumul	5.	8.89	{2., 19.}	119.8	0.339477
44	newly 3 weeks spec	3.	1.67	{0., 6.}	102.333	1.10086	newly 3 weeks cumul	3.	10.88	{2., 28.}	282.	0.46705	newly 5 weeks spec	0.1	0.27	{0., 0.}	358.	0.0900346	newly 5 weeks cumul	3.	11.88	{2., 28.}	315.333	0.479756	newly 44 weeks cumul	3.	12.17	{2., 28.}	325.	0.481789
45	newly 3 weeks spec	7.	3.37	{0., 10.}	75.2857	0.921122	newly 3 weeks cumul	21.	16.35	{4., 31.}	47.	0.30734	newly 5 weeks spec	2.	1.5	{0., 5.}	120.	1.00219	newly 5 weeks cumul	25.	20.03	{4., 40.}	52.36	0.339598	newly 42 weeks cumul	30.	22.02	{4., 42.}	57.2	0.389638
46	newly 3 weeks spec	13.	9.08	{0., 19.}	56.7692	0.546623	newly 3 weeks cumul	34.	31.35	{9., 61.}	46.2059	0.257162	newly 5 weeks spec	14.	6.09	{0., 18.}	70.7857	0.974712	newly 5 weeks cumul	70.	44.97	{10., 94.}	50.1571	0.370605	newly 49 weeks cumul	231.	63.41	{10., 127.}	72.5498	0.740905
47	newly 3 weeks spec	8.	8.37	{0., 18.}	64.625	0.437267	newly 3 weeks cumul	29.	29.11	{9., 54.}	43.	0.218046	newly 5 weeks spec	9.	6.85	{0., 17.}	77.8889	0.779313	newly 5 weeks cumul	47.	43.34	{12., 82.}	46.8085	0.261073	newly 47 weeks cumul	220.	72.54	{12., 154.}	67.7182	0.659554
48	newly 3 weeks spec	9.	3.29	{0., 12.}	88.7778	1.41404	newly 3 weeks cumul	23.	33.27	{3., 67.}	98.7391	0.415895	newly 5 weeks spec	13.	0.41	{0., 0.}	97.3077	2.0278	newly 5 weeks cumul	45.	34.75	{3., 82.}	61.8444	0.463656	newly 42 weeks cumul	52.	34.78	{3., 82.}	60.4231	0.495569
49	newly 3 weeks spec	14.	6.99	{0., 14.}	59.6429	0.660725	newly 3 weeks cumul	26.	26.4	{7., 49.}	48.9231	0.253778	newly 5 weeks spec	13.	4.74	{0., 15.}	74.4615	1.1037	newly 5 weeks cumul	51.	36.99	{8., 74.}	48.6471	0.344784	newly 46 weeks cumul	150.	57.12	{8., 135.}	64.9467	0.637835
50	newly 3 weeks spec	9.	5.21	{0., 11.}	65.8889	0.754384	newly 3 weeks cumul	30.	27.47	{8., 46.}	45.5	0.256909	newly 5 weeks spec	4.	2.43	{0., 7.}	96.75	1.10483	newly 5 weeks cumul	48.	33.77	{8., 69.}	49.5208	0.343557	newly 43 weeks cumul	70.	39.05	{8., 87.}	58.8143	0.459237
